# CpxR Activates MexAB-OprM Efflux Pump Expression and Enhances Antibiotic Resistance in Both Laboratory and Clinical *nalB*-Type Isolates of *Pseudomonas aeruginosa*

**DOI:** 10.1371/journal.ppat.1005932

**Published:** 2016-10-13

**Authors:** Zhe-Xian Tian, Xue-Xian Yi, Anna Cho, Fergal O’Gara, Yi-Ping Wang

**Affiliations:** 1 State Key Laboratory of Protein and Plant Gene Research, College of Life Sciences, Peking University, Beijing, China; 2 BIOMERIT Research Centre, Department of Microbiology, University College Cork, Cork, Ireland; 3 School of Biomedical Sciences, Curtin Health Innovation Research Institute, Curtin University, Perth, Western Australia, Australia; University of Washington, UNITED STATES

## Abstract

Resistance-Nodulation-Division (RND) efflux pumps are responsible for multidrug resistance in *Pseudomonas aeruginosa*. In this study, we demonstrate that CpxR, previously identified as a regulator of the cell envelope stress response in *Escherichia coli*, is directly involved in activation of expression of RND efflux pump MexAB-OprM in *P*. *aeruginosa*. A conserved CpxR binding site was identified upstream of the *mexA* promoter in all genome-sequenced *P*. *aeruginosa* strains. CpxR is required to enhance *mexAB-oprM* expression and drug resistance, in the absence of repressor MexR, in *P*. *aeruginosa* strains PA14. As defective *mexR* is a genetic trait associated with the clinical emergence of *nalB*-type multidrug resistance in *P*. *aeruginosa* during antibiotic treatment, we investigated the involvement of CpxR in regulating multidrug resistance among resistant isolates generated in the laboratory via antibiotic treatment and collected in clinical settings. CpxR is required to activate expression of *mexAB-oprM* and enhances drug resistance, in the absence or presence of MexR, in ofloxacin-cefsulodin-resistant isolates generated in the laboratory. Furthermore, CpxR was also important in the *mexR*-defective clinical isolates. The newly identified regulatory linkage between CpxR and the MexAB-OprM efflux pump highlights the presence of a complex regulatory network modulating multidrug resistance in *P*. *aeruginosa*.

## Author Summary

*Pseudomonas aeruginosa* is one of the major pathogens associated with cystic fibrosis and multidrug resistant *P*. *aeruginosa* has been listed as the Top 10 antibiotic resistance threats in the US CDC report (http://www.cdc.gov/drugresistance/biggest_threats.html). Drug efflux systems play a major role in multidrug resistance in *P*. *aeruginosa*. Currently, the regulatory networks modulating efflux pump expression are not fully understood. Here, we demonstrate that CpxR, a potentially multifaceted regulator, is directly involved in regulation of expression of MexAB-OprM, the major efflux pump in *P*. *aeruginosa*. The newly identified activator CpxR plays an important role in modulating multidrug resistance in *nalB*-type laboratory and clinical isolates. This work provides insight into the complex regulatory networks modulating multidrug resistance in *P*. *aeruginosa*.

## Introduction

*Pseudomonas aeruginosa*, a major pathogen associated with cystic fibrosis, is known for its intrinsic resistance to a wide range of antimicrobial agents and its ability to develop multidrug resistance following antibiotic therapy [1]. Resistance-Nodulation-Division (RND) efflux systems are largely responsible for intrinsic and acquired multidrug resistance in *P*. *aeruginosa*; genes encoding 12 RND efflux pumps have been identified in its genome [2, 3].

Genes encoding RND efflux pumps are highly conserved in the genomes of many living organisms [4]. Recently, increasing attention has been focused on the physiological roles of efflux pumps relevant to the behaviour of bacteria in their natural ecosystems [4–6]. Accumulating evidence has demonstrated that efflux pumps are also important for processes of detoxification of intracellular metabolites, bacterial virulence in animal and plant hosts, cell homeostasis, and intercellular signalling [4]. Previously, we identified a novel MexT regulon, incorporating the MexEF-OprN efflux pump into a broader physiological context in *P*. *aeruginosa* [7]. MexT binding sites in the promoter regions of MexT regulon genes in *P*. *aeruginosa* are conserved in the promoter regions of orthologous MexT regulon genes in other *Pseudomonas* species. It is generally accepted that divergence of regulatory sites is slower than that of most non-coding regions among closely related species. This concept has been used to identify novel regulatory sites by comparing the promoter regions of orthologous RND efflux pump genes from closely related species [8].

The MexAB-OprM efflux pump plays a significant role in multidrug resistance in *P*. *aeruginosa* [2, 3]. Overexpression of the *mexAB-oprM* operon was first identified in *nalB*-type *P*. *aeruginosa* strains, a phenotypic group showing multiple antibiotic resistance [9]. It is now known that two tandem promoters control expression of the *mexAB-oprM* operon in *P*. *aeruginosa*; the distal promoter is modulated by repressor MexR [10, 11], while the proximal promoter is modulated by repressor NalD [12]. A third repressor, NalC, indirectly modulates expression of the *mexAB-oprM* operon by controlling the expression level of ArmR, an anti-MexR protein [13–15]. Mutations causing defective forms of MexR, NalC, and NalD lead to overexpression of the *mexAB-oprM* operon and enhance multidrug resistance in *P*. *aeruginosa* [10–14]. In particular, mutations in *mexR* are the major genotypes associated with *nalB*-type strains and are often identified among clinical isolates [10–12, 16, 17]. In addition to MexR, NalC, and NalD, additional regulatory components have been shown to influence expression of the *mexAB-oprM* operon in *P*. *aeruginosa*. MexT, a LysR-type activator of RND efflux pump MexEF-OprN, exerts a negative regulatory effect on MexAB-OprM expression through an uncharacterized mechanism in *nfxC*-type *P*. *aeruginosa* strains [18]. RocA2, a response regulator of the pilus assembly machinery cluster operon, also exerts a negative regulatory effect on MexAB-OprM expression, indicating a potential functional linkage between the MexAB-OprM efflux pump and biofilm formation [19]. BrlR, a biofilm-specific MerR-type regulator, activates MexAB-OprM expression through its binding to the promoter region during biofilm formation in *P*. *aeruginosa* [20]. AmpR, a LysR-type regulator of AmpC β-lactamase, also exerts a positive regulatory effect on MexAB-OprM expression by repressing MexR expression [21]. The existence of multiple regulatory components renders the *mexAB-oprM* operon subject to complex regulation in *P*. *aeruginosa*.

As a response regulator, CpxR was first identified as an important regulator for protecting cell envelope and promoting cell survival in *Escherichia coli* [22–24]. Numerous studies have verified the role of CpxR in antibiotic resistance in pathogenic bacteria. In *E*. *coli*, overexpression of CpxR confers resistance to β-lactams in a drug-hypersusceptible mutant, in which AcrAB, a major efflux pump, was defective [25]; CpxR is involved in the defence response to aminoglycoside-induced oxidative stress [26, 27]; it confers resistance to fosfomycin by directly repressing *glpT* and *uhpT* expression in enterohemorrhagic *E*. *coli* [28]; Induction of the CpxR pathway directly contributes to tolerance toward certain antimicrobial peptides, including polymyxin B and protamine [29, 30]. In *Salmonella typhimurium*, CpxR also confers resistance to antimicrobial peptides protamine, magainin, and melittin through activation of two Tat-dependent peptidoglycan amidases [31]; moreover, it confers strong ceftriaxone resistance by modulating expression of *STM1530* and *ompD* [32]; Laboratory-generated and clinical *S*. *typhimurium* strains lacking CpxR show reduced resistance to aminoglycosides and β-lactams [33]. In *Klebsiella pneumoniae*, CpxR is involved in multidrug resistance through direct promoter binding and activation of *ompC* and *kpnEF* [34, 35]. In *Vibrio cholerae*, CpxR can activate expression of RND efflux pumps VexAB and VexGH, which can confer resistance to ampicillin [36]. *Erwinia amylovora* lacking CpxR show reduced resistance to β-lactams, aminoglycosides, and lincomycin [37]. Although CpxR is widely distributed in the genomes of various gamma-proteobacteria, its role in *Pseudomonas* species remains unknown.

In this study, bioinformatics, biochemical, and genetic analyses identified a regulatory linkage between CpxR and multidrug efflux pump MexAB-OprM in *P*. *aeruginosa*. We show that CpxR activates *mexAB-oprM* expression by directly binding to the distal promoter and is important for multidrug resistance in *nalB*-type *P*. *aeruginosa* isolates under both laboratory and clinical conditions.

## Results

### Comparative genomic analysis of RND efflux pumps

**Fig 1:**
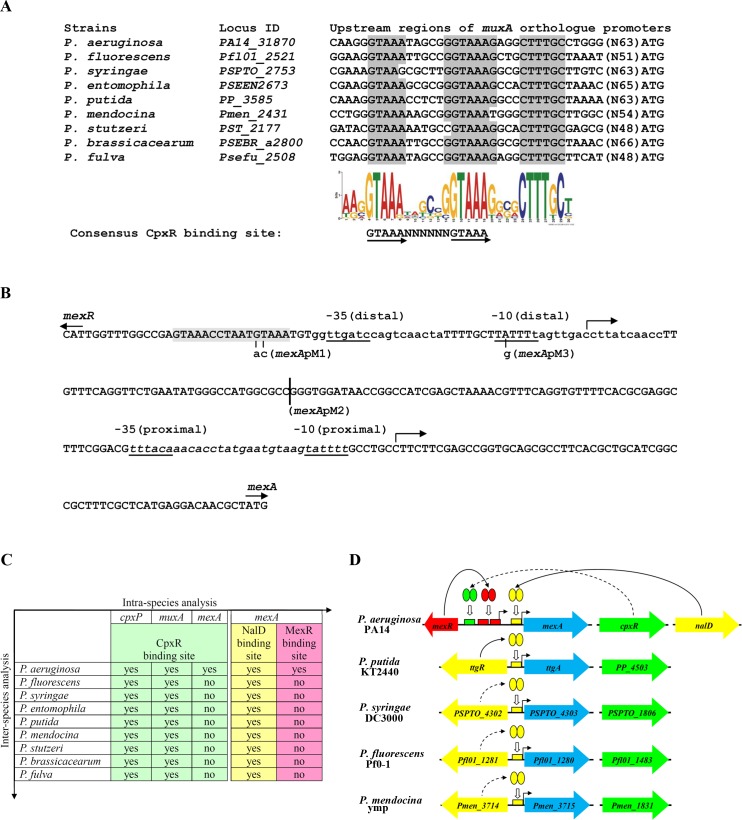
Comparative genomic analysis of conserved DNA motifs. (A) A well-conserved DNA motif exists in the promoter regions of orthologous *muxABC-opmB* operons in *Pseudomonas* species. The sequence logo for the conserved DNA motif reflects position-specific probability matrixes; high probability (≥ 70%) nucleotides are marked in grey in the alignment. The DNA motif contains a well-conserved CpxR binding site. The number in the blanket is the distance between the DNA motif and the ATG start codon of each gene locus. (B) DNA sequence of the *mexR-mexA* intergenic region. The ATG start codons of *mexA* and *mexR* are under solid arrows, indicating the directions of the coding sequences. The -35 and -10 regions of the distal and proximal promoters of *mexA* are underlined [12, 40]. The transcriptional start sites of the distal and proximal promoters of *mexA* are indicated by bent arrows [12, 40]. The putative CpxR binding site is shaded, whereas the two MexR binding sites overlapping the -35/-10 region of the distal promoter are indicated in lower-case letters [40, 41]. A NalD binding site overlapping the -35/-10 region of the proximal promoter is indicated in italic lower-case letters [12]. The nucleotide substitutions for the mutated *mexA* promoters (*mexA*p_M1_ and *mexA*p_M3_) are paired with a short vertical line. The downstream deletion boundary of the distal-only *mexA* promoter (*mexA*p_M2_) is indicated with a long vertical line. (C) A well-conserved CpxR binding site exists in the promoter region of each *cpxP* and *muxA* orthologue, while a well-conserved NalD binding site exists in the promoter region of each *mexA* orthologue. In contrast, the conserved CpxR and MexR binding site on the *mexA* promoter is unique to *P*. *aeruginosa*. (D) Components that might be involved in regulating *mexA* orthologue (in blue) expression in *Pseudomonas* species. The orthologous loci of *cpxR* and *nalD* among different *Pseudomonas* species are marked in green and yellow, respectively. The orthologous *nalD* locus is separated and replaced by the *mexR* locus in *P*. *aeruginosa*, but divergently linked to the orthologous *mexA* locus in the genomes of other *Pseudomonas* species.

In order to unravel the regulatory networks responsible for modulating the expression of RND efflux pumps in *P*. *aeruginosa*, comparative genomic analysis was carried out to identify novel regulatory sites on the promoters of orthologous RND operons among different *Pseudomonas* species. In this case, we compared the promoter regions of the orthologous operons of *mexAB-oprM*, *mexEF-oprN*, and *muxABC-opmB* in 15 genome-sequenced *Pseudomonas* species. The results showed that, apart from the previously identified NalD repressor binding site on the *mexA* promoter [12] (see S1A Fig), a well conserved DNA motif was identified on the *muxA* promoter (Fig 1A). Interestingly, the motif contains a consensus binding site (5′-GTAAA-(N)_4-8_-GTAAA-3′) for CpxR, a response regulator of the two-component system in *E*. *coli* [38]. The gene locus *PA14_22760* has been annotated as *cpxR* in the genome of *P*. *aeruginosa* strain PA14 in the Pseudomonas database [39]; it encodes a protein with the highest BLASTP score (47% identity) with *E*. *coli* CpxR among the ORFs of *P*. *aeruginosa* strain PA14.

As CpxR is a global regulator of the cell envelope stress response in *E*. *coli* [22, 42] and might regulate the *muxABC-opmB* operon (as shown in the inter-species analysis above; Fig 1A), we used its binding site (5′-GTAAA-(N)_4-8_-GTAAA-3′) as a probe to perform intra-species analysis of the genome of *P*. *aeruginosa* PA14. Because CpxR can exert its activity independent of the orientation of its binding site [38], the existence of potential CpxR binding sites on both strands was assessed. The results showed that a number of genes possess the consensus CpxR binding site on their promoter regions (S1 Table). Among such genes, *PA14_22740*, which is adjacent to the *cpxR* locus in the genome of *P*. *aeruginosa* strain PA14, encodes a small, putative periplasmic protein with two LTXXQ motifs (S2 Fig), a canonical feature of the protein encoded by *cpxP*, the cognate target gene of CpxR in *E*. *coli* [43, 44]. Furthermore, among *P*. *aeruginosa* strain PA14 genes, the protein encoded by *PA14_22740* showed the highest BLASTP score (25% identity) with *E*. *coli* CpxP protein. Thus, we annotated *PA14_22740* as a *cpxP* gene in *P*. *aeruginosa* strain PA14. Surprisingly, the promoter of *mexAB-oprM* in *P*. *aeruginosa* PA14 also contains a consensus CpxR binding site (S1 Table and Fig 1B).

The inter-species analysis showed that the conserved CpxR binding site is present on the promoter regions of the identified *cpxP* orthologues, similar to the case of the *muxA* orthologues (Fig 1C, for the details see S1B Fig). In contrast, the presence of the CpxR binding site on the *mexA* promoter is unique to *P*. *aeruginosa* among the 15 *Pseudomonas* species for which the entire genome has been sequenced. Therefore, for the first time, by using comparative genomic analysis, we have found a potential regulatory linkage between CpxR and *mexAB-oprM* in *P*. *aeruginosa*.

Since the newly identified CpxR binding site is located upstream of the distal promoter of *mexA*, which is known to be modulated by the MexR repressor, we further investigated the existence of the MexR binding site (5′-GTTGA-(N)_5_-TCAAC-3′, Fig 1B) [40, 41] in the promoter regions of *mexA* orthologues among *Pseudomonas* species. The results showed that the presence of the MexR binding site on the *mexA* promoter is unique to *P*. *aeruginosa* (Fig 1C). In contrast, the NalD binding site is well conserved in the promoter region of each *mexA* orthologue (Fig 1C and S1A Fig). In fact, the *nalD* orthologue (*ttgR*) locus is divergently linked to the *mexA* orthologue locus in the genomes of other *Pseudomonas* species, while the *mexR* locus is divergently linked to the *mexA* locus, which is completely separated from the *nalD* locus in the genome of *P*. *aeruginosa* (Fig 1D). These observations indicate a species-specific coupling of multiple regulators (CpxR and MexR) with the MexAB-OprM efflux pump in *P*. *aeruginosa*.

### Direct activation of *mexAB-oprM* expression by CpxR

When the *mexA*, *muxA*, and *cpxP* promoters were fused with the *lacZ* reporter gene and their expression levels were monitored, we found that their activities were under the control of CpxR in *P*. *aeruginosa*. In particular, expression of the *mexA*, *muxA*, and *cpxP* promoters was strongly activated by the presence of ectopically expressed CpxR in PA14Δ*cpxR* (Table 1). In contrast, when the newly identified CpxR binding site on the *mexA* promoter was altered by site-directed mutagenesis (*mexA*p_M1_, for details see Fig 1B), CpxR-dependent activation was completely abolished. When the conserved phosphorylation site (the 52^nd^ aspartate residue) of CpxR was mutated to alanine (CpxR^D52A^), the ectopically expressed CpxR could not activate expression of target promoters in PA14Δ*cpxR* (Table 1). As the stability of CpxR^D52A^ is not altered in comparison with that of wild-type CpxR (S3 Fig), these results suggest that phosphorylation of CpxR is important for its activity.

**Table 1:** CpxR activates expression of target promoters.

Reporter system	β-galactosidase activity (Miller units)		
	Vector	pCpxR	pCpxR^D52A^
*P*. *aeruginosa* PA14Δ*cpxR* background			
*cpxP*p::*lacZ*	42 ± 2	5245 ± 170	41 ± 10
*muxA*p::*lacZ*	34 ± 1	1613 ± 10	38 ± 1
*mexA*p::*lacZ*	125 ± 2	1594 ± 138	117 ± 5
*mexA*p_M1_::*lacZ*	96 ± 3	81 ± 12	97 ± 6
*mexA*p_M2_::*lacZ*	88 ± 1	1140 ± 78	147 ± 1
*mexA*p_M3_::*lacZ*	63 ± 3	51 ± 13	72 ± 3
*P*. *putida* KT2440 background			
*PP4504*p::*lacZ*	43 ± 4	1826 ± 74	NA
*PP3585*p::*lacZ*	66 ± 3	307 ± 25	NA
*ttgA*p::*lacZ*	36 ± 3	42 ± 5	NA

NA, not assayed.

**Fig 2:**
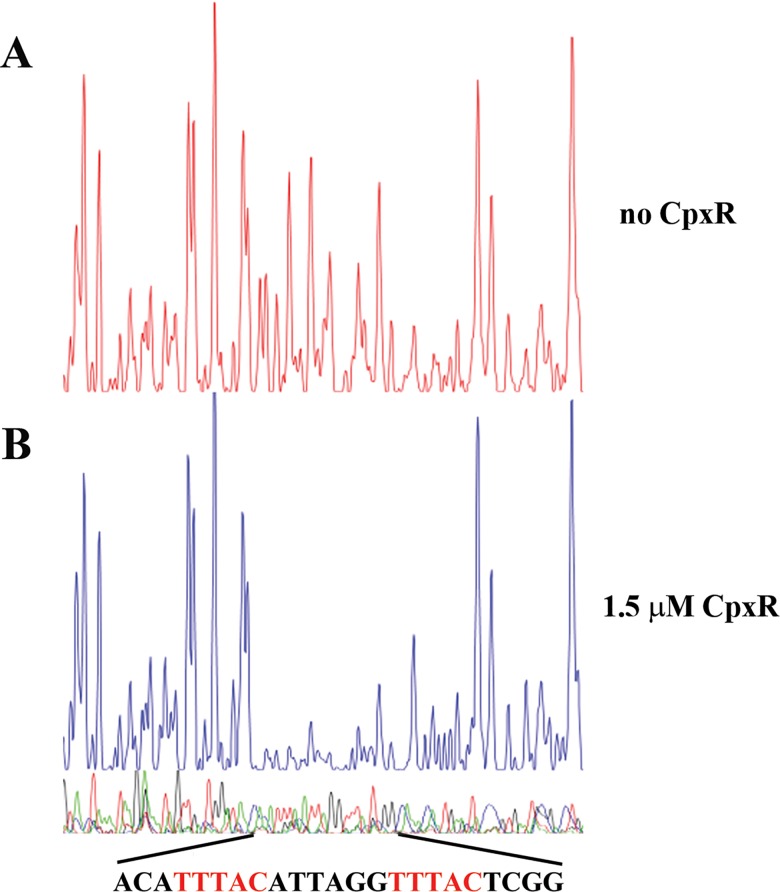
Direct binding of CpxR to the promoter region of *mexA in vitro*. DNase I footprinting assays of the *mexA* promoter DNA fragment were performed in the absence (A) and presence (B) of purified CpxR. The FAM-labelled 322-bp DNA fragments (50 nM) pre-incubated in the absence or presence of 1.5 μM phosphorylated CpxR were subjected to DNase I digestion and fragment length analysis. The fluorescence signal of the labelled DNA fragments is plotted against the sequence of the fragment. The protected region bound by CpxR is shown with the conserved binding motif in red.

The importance of phosphorylation in CpxR activation is further supported by the fact that the phosphorylated form of CpxR clearly bound to the target promoter region containing the intact conserved DNA binding site in a concentration-dependent manner in electrophoretic mobility shift assays (EMSAs) (S4A and S4B Fig, from lane 2 to 5). In contrast, such binding was abolished when an excess amount of unlabelled competitor DNA was present or the non-phosphorylated form of CpxR (in the absence of acetyl phosphate) was used in the assay (S4A and S4B Fig, lane 6 and 7 respectively). When DNA fragments with a mutated CpxR binding site were used in the assay, no binding was observed (S4C Fig). DNase I footprinting analysis further confirmed the location of the binding site of phosphorylated CpxR in the *mexA* promoter region (Fig 2A and 2B). Taken together, these results demonstrate that CpxR binds directly to its conserved DNA binding site in a phosphorylation-dependent manner, and such binding is essential for CpxR-dependent activation of the target promoters in *P*. *aeruginosa*.

The unique *P*. *aeruginosa*-specific regulatory linkage between CpxR and MexAB-OprM was demonstrated by similar experiments in an alternative host, *Pseudomonas putida* KT2440. In this strain, consensus CpxR binding sites exist on the promoters of *PP_4504* and *PP_3585*, the orthologous genes of *cpxP* and *muxA*, respectively, but not on the promoter of *ttgA*, the orthologous gene of *mexA*. In *P*. *putida* KT2440, ectopically expressed CpxR significantly activates expression of *PP_4504* and *PP_3585*, but does not alter expression of *ttgA* (Table 1). Therefore, among the *Pseudomonas* species analysed, CpxR-dependent activation of the promoter of *mexAB-oprM* is unique in *P*. *aeruginosa*.

As CpxR can activate expression of *mexAB-oprM* and *muxABC-opmB*, the contributions of these genes to multidrug resistance in *P*. *aeruginosa* were investigated. Minimal inhibitory concentrations (MICs) of ciprofloxacin, ofloxacin, ceftazidime, cefsulodin, and aztreonam, but not amikacin, were increased at least 4-fold by ectopically expressed CpxR in PA14 and PA14Δ*cpxR* strains (Table 2) in a manner dependent on MexA, but not MuxA. In this case, ectopically expressed CpxR failed to increase the MICs of the tested antibiotics in a *mexA* null-mutant PA14Δ*mexA* strain. In contrast, the MIC increases caused by the ectopically expressed CpxR were not altered in a *muxA* null-mutant PA14Δ*muxA* strain (Table 2). These results indicate that CpxR activates expression of *mexAB-oprM*, which enhances multidrug resistance in *P*. *aeruginosa*.

**Table 2:** CpxR-mediated enhancement of multidrug resistance in *P*. *aeruginosa* is MexA-dependent, but not MuxA-dependent.

	MIC (μg/mL)					
Strain	Cip	Ceft	Ofl	Cefs	Azt	Amk
PA14+ vector	0.13	1.0	0.5	1.0	4.0	2.0
PA14+ pCpxR	0.5	4.0	4.0	4.0	16	2.0
PA14Δ*cpxR* + vector	0.13	1.0	0.5	1.0	4.0	2.0
PA14Δ*cpxR* + pCpxR	0.5	4.0	4.0	4.0	16	2.0
PA14Δ*mexA* + vector	0.06	0.5	0.13	0.5	1.0	2.0
PA14Δ*mexA* + pCpxR	0.06	0.5	0.13	0.5	1.0	2.0
PA14Δ*muxA* + vector	0.13	1.0	0.5	1.0	4.0	2.0
PA14Δ*muxA* + pCpxR	0.5	4.0	4.0	4.0	16	2.0

Cip, ciprofloxacin; Ceft, ceftazidime; Ofl, ofloxacin; Cefs, cefsulodin; Azt, aztreonam; Amk, amikacin

The newly identified CpxR binding site is located upstream of the distal promoter of *mexA* in *P*. *aeruginosa*. To determine which promoter (distal or proximal) is activated by CpxR, two *mexA* promoter-*lacZ* reporter systems were constructed. To monitor the expression of the distal promoter, the entire proximal promoter region was excluded in the *mexA*p_M2_::*lacZ* construct; to monitor the expression of the proximal promoter, a key nucleotide within the -10 region of the distal promoter [45] was disrupted in the *mexA*p_M3_::*lacZ* construct (Fig 1B). Ectopically expressed CpxR strongly activated expression of *mexA*p_M2_::*lacZ*, but not *mexA*p_M3_::*lacZ*, in the PA14Δ*cpxR* strain (Table 1), indicating that CpxR is involved only in regulation of the distal *mexA* promoter, which is also directly regulated by the MexR repressor in *P*. *aeruginosa*.

**Fig 3:**
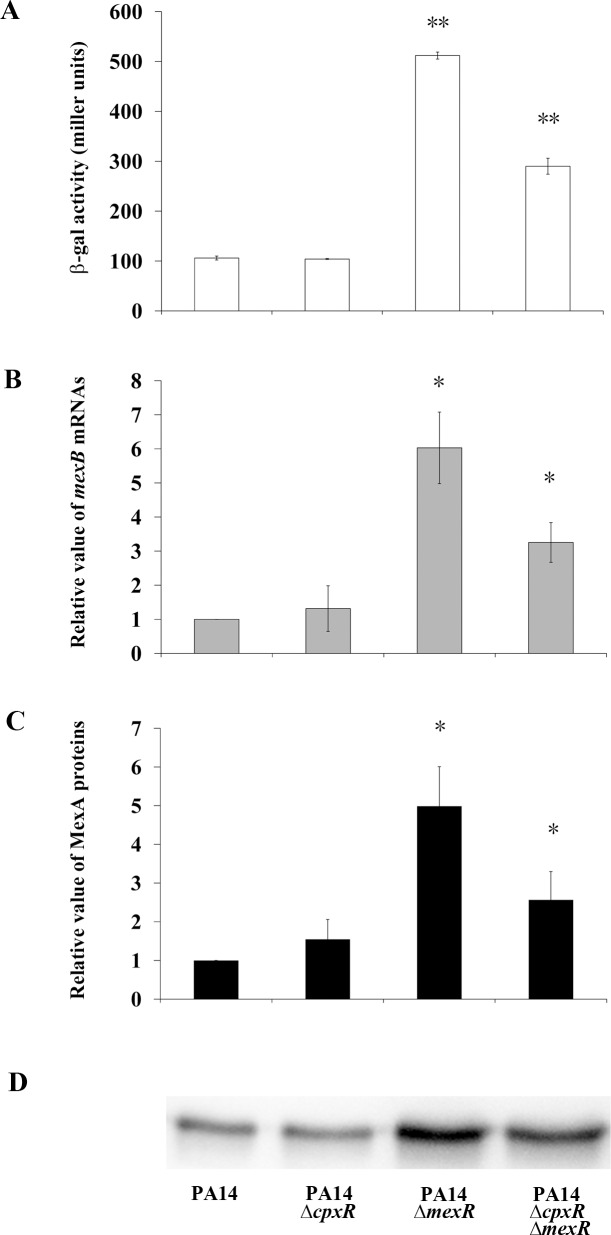
CpxR-mediated up-regulation of *mexAB-oprM* expression in *mexR*-deleted *P*. *aeruginosa* PA14. (A) The expression levels of *mexA*p::*lacZ* reporter as measured by β-galactosidase assay in the PA14, PA14Δ*cpxR*, PA14Δ*mexR*, and PA14Δ*cpxR*Δ*mexR* strains. Each bar represents the mean ± SD of two independent experiments (**, *p* < 0.01). (B) Relative *mexB* transcript levels determined by quantitative real-time PCR in the PA14, PA14Δ*cpxR*, PA14Δ*mexR*, and PA14Δ*cpxR*Δ*mexR* strains. Data are expressed relative to the quantity of *mexB* mRNA in the wild-type PA14 strain. Each bar represents the mean ± SD of the relative quantity in three independent experiments (*, *p* < 0.05). (C) Relative MexA protein levels were determined by western blotting in the PA14, PA14Δ*cpxR*, PA14Δ*mexR*, and PA14Δ*cpxR*Δ*mexR* strains. The intensity of each band was quantified. The results are expressed relative to the quantity of MexA in the wild-type PA14 strain. Each bar represents the mean ± SD of the relative quantity in three independent experiments (*, *p* < 0.05). (D) A representative western blot image of MexA protein in the PA14, PA14Δ*cpxR*, PA14Δ*mexR*, and PA14Δ*cpxR*Δ*mexR* strains. The membrane proteins (10 μg per lane) were separated by 10% SDS-PAGE and immunoblotted with anti-MexA polyclonal antibodies.

The possible interplay between CpxR and MexR on expression of the distal *mexA* promoter prompted us to investigate the involvement of CpxR in the multidrug resistance phenotype of *nalB*-type *P*. *aeruginosa*, which has been associated with defective MexR in laboratory and clinical isolates [13, 16, 17]. We monitored *mexA* expression levels in strains of various genetic backgrounds, including *mexR* null-mutant PA14Δ*mexR*, *cpxR* null-mutant PA14Δ*cpxR*, *cpxR/mexR* double-mutant PA14Δ*cpxR*Δ*mexR*, and wild-type PA14 strains. The *mexA* expression level of the PA14Δ*mexR* strain was significantly higher than that of the wild-type PA14 strain (Fig 3), a result similar to that previously reported for *nalB*-type *P*. *aeruginosa* [12]. Moreover, the lack of CpxR in the PA14Δ*cpxR*Δ*mexR* strain resulted in decreased *mexA* expression (Fig 3). To further confirm the regulatory influence of CpxR on expression of MexAB-OprM, the relative transcript level of *mexB* and protein level of MexA were investigated in the wild-type and mutant strains by quantitative real-time PCR and western blot analysis, respectively. The regulatory patterns of CpxR on the expression of the chromosomal genes were similar to that of the *mexA*p::*lacZ* reporter system in the tested strains (Fig 3).

To evaluate the influence of changes in *mexA* expression on drug resistance, the MICs of antibiotics were determined. In the PA14Δ*mexR* strain, the MICs of the antibiotics were significantly increased in comparison with those of the parental PA14 strain. Moreover, a lack of *cpxR* led to decreased MICs in the PA14Δ*cpxR*Δ*mexR* strain in comparison with those of the PA14Δ*mexR* strain. These results indicate that the MICs of antibiotics in the tested strains are correlated well with their expression levels of *mexA* (Table 3). A similar effect was observed when *cpxR* (*PA3204*) was deleted in a *mexR*-deleted mutant of another standard laboratory *P*. *aeruginosa* strain, PAO1 (PAO1Δ*mexR*), indicating that the observed effect was not specific to a particular *P*. *aeruginosa* strain (Table 3).

**Table 3:** CpxR mediates enhancement of antibiotic resistance in *mexR*-deleted *P*. *aeruginosa* laboratory standard strains.

	MIC (μg/mL)				
Strain	Cip	Ceft	Ofl	Cefs	Azt
PA14	0.13	1.0	0.5	1.0	4.0
PA14Δ*cpxR*	0.13	1.0	0.5	1.0	4.0
PA14Δ*mexR*	0.5	4.0	4.0	4.0	16
PA14Δ*cpxR*Δ*mexR*	0.25	2.0	2.0	2.0	8.0
PAO1	0.13	1.0	0.5	1.0	4.0
PAO1Δ*cpxR*	0.13	1.0	0.5	1.0	4.0
PAO1Δ*mexR*	0.5	4.0	2.0	4.0	16
PAO1Δ*cpxR*Δ*mexR*	0.25	2.0	1.0	2.0	8.0

### CpxR activates expression of *mexAB-oprM* and enhances multidrug resistance in *nalB*- type *P. aeruginosa* resistant isolates from the laboratory and a clinical setting

Defective *mexR* is a genetic trait associated with the clinical emergence of multidrug resistance in *P*. *aeruginosa* during antibiotic treatment [16]. Previously, *mexR* defective strains were selected *in vitro* by plating susceptible *P*. *aeruginosa* strains on agar medium containing lethal levels of a fluorquinolone antibiotic (ofloxacin or ciprofloxacin) and a third-generation cephalosporin antibiotic (cefsulodin or cefoperazone) [10, 11, 46, 47]. In this work, PA14 cells were plated on agar medium containing lethal levels of ofloxacin and cefsulodin antibiotics, after which 40 ofloxacin-cefsulodin resistant (OCR) colonies were randomly collected for further analysis. Among the selected colonies, five isolates exhibited significantly reduced expression levels of *mexA*, as well as reduced MICs of ciprofloxacin, ceftazidime, and aztreonam, when *cpxR* was deleted (Table 4). When the *mexR* sequences of the five isolates were analysed, PA14OCR16, PA14OCR24, PA14OCR28, and PA14OCR32 were found to harbour defective mutations. The plasmid harbouring *cpxR*, but not *cpxR* ^D52A^, complemented the phenotype, indicating that CpxR mediated the observed alteration in isolate PA14OCR16 (Table 4). The pattern of CpxR-dependent activation of *mexAB-oprM* expression and enhancement of multidrug resistance in the four OCR isolates with defective mutations in *mexR* was identical to that of the engineered PA14Δ*mexR* strain (Table 3), implying that CpxR might perform a common function in *mexR*-defective *nalB*-type *P*. *aeruginosa* strains.

**Table 4:** CpxR mediates up-regulation of *mexAB-oprM* expression levels and enhancement of antibiotic resistance in isolated *nalB-*type *P*. *aeruginosa* strains *in vitro*.

Strain	*mexR* locus	β-galactosidase activity (Miller units)		MIC (μg/mL)		
		*mexA*p::*lacZ*	*cpxP*p::*lacZ*	Cip	Ceft	Azt
PA14	Wild-type	106 ± 4	60 ± 3	0.13	1.0	4.0
PA14Δ*cpxR*		104 ± 1	24 ± 1	0.13	1.0	4.0
PA14OCR16	Insertion of T after A_80_ ^a^	490 ± 16	67 ± 5	0.5	4.0	16
PA14OCR16Δ*cpxR*		228 ± 6	34 ± 1	0.25	2.0	8.0
PA14OCR16Δ*cpxR* (pCpxR)		864 ± 42	337 ± 21	0.5	4.0	16
PA14OCR16Δ*cpxR* (pCpxR^D52A^)		275 ± 28	86 ± 5	0.25	2.0	8.0
PA14OCR24	Insertion of T after A_80_	507 ± 12	56 ± 1	0.5	4.0	16
PA14OCR24Δ*cpxR*		250 ± 2	30 ± 1	0.25	2.0	8.0
PA14OCR28	Deletion of C_110_	474 ± 6	71 ± 2	0.5	4.0	16
PA14OCR28Δ*cpxR*		225 ± 15	32 ± 2	0.25	2.0	8.0
PA14OCR32	Deletion of C_32_~C_55_	492 ± 7	58 ± 2	0.5	4.0	16
PA14OCR32Δ*cpxR*		226 ± 11	30 ± 1	0.25	2.0	8.0
PA14OCR33^b^	Intact	264 ± 11	57 ± 8	0.25	2.0	8.0
PA14OCR33Δ*cpxR*		231 ± 9	30 ± 6	0.25	2.0	8.0
PA14OCR36	Intact	597 ± 17	1558 ± 78	0.5	4.0	16
PA14OCR36Δ*cpxR*		121 ± 1	30 ± 3	0.13	1.0	4.0
PA14OCR36Δ*cpxR* (pCpxR)		577 ± 44	1069 ± 17	0.5	4.0	16
PA14OCR36Δ*cpxR* (pCpxR^D52A^)		109 ± 2	61 ± 1	0.13	1.0	4.0

^a^ This genotype has also appeared among previously *in vitro*-selected *nalB* type *P*. *aeruginosa* strains [11].

^b^ This strain shows the *nalD* phenotype with a frameshift mutation in the *nalD* locus caused by deletion of C_486_.

Interestingly, the fifth OCR isolate, PA14OCR36, had an intact *mexR* gene. In this particular isolate, the expression level of *cpxP* was drastically increased with respect to those of *mexR*-defective isolates PA14OCR16, PA14OCR24, PA14OCR28, and PA14OCR32. In parallel, the *mexA* expression level of isolate PA14OCR36 was comparable to those of the *mexR*-defective strains (Table 4). Deletion of *cpxR* from isolate PA14OCR36 resulted in drastically decreased expression levels of *cpxP* and *mexA*. The plasmid harbouring *cpxR*, but not *cpxR* ^D52A^, complemented the phenotype, indicating that CpxR is important for the observed alteration in *cpxP* expression in isolate PA14OCR36 (Table 4). Sequence analysis indicated the *cpxR*, *nalC*, and *nalD* genes of isolate PA14OCR36 were intact, suggesting that this isolate was distinct from constitutively active CpxR mutants or previously known *nalC*- or *nalD*-type mutants [12, 14]. These results indicate that CpxR could override repression by MexR upon expression of the *mexAB-oprM* operon in isolate PA14OCR36, a newly identified *nalB*-phenotype OCR isolate of *P*. *aeruginosa*.

OCR isolate PA14OCR33 has a null mutation in the *nalD* locus and elevated *mexA* expression; *mexA* expression in PA14OCR33 is independent of CpxR, because deletion of *cpxR* did not alter the expression level of *mexA* in this strain (Table 4). This result confirms that CpxR plays no role in the regulatory influence of the NalD repressor on the expression of *mexAB-oprM* in *P*. *aeruginosa*.

To evaluate the importance of CpxR under clinical conditions, we obtained *P*. *aeruginosa* clinical isolates from the Department of Microbiology, Chinese People’s Liberation Army General Hospital (Beijing, China). Fifty independent clinical isolates exhibiting ciprofloxacin and ceftazidime resistance were analysed, among which three isolates, LAR005, LAR023, and LAR048, exhibited significantly reduced expression levels of *mexA*, as well as reduced MICs of ciprofloxacin, ceftazidime and aztreonam, when *cpxR* was deleted (Table 5). When the *mexR* sequences of clinical isolates LAR005, LAR023, and LAR048 were analysed, each was found to harbour frame-shifted or nonsense mutations at different sites in the *mexR* coding region (Table 5). Taken together, these results indicate that CpxR plays an important role in modulating multidrug resistance in *nalB*-type *P*. *aeruginosa* isolates generated in the laboratory and collected in the clinic.

**Table 5:** CpxR mediates up-regulation of *mexAB-oprM* expression levels and enhancement of antibiotic resistance in *mexR-*defective *P*. *aeruginosa* clinical isolates.

Strain		*mexR* locus	β-galactosidase activity (Miller units)	MIC (μg/mL)		
			*mexA*p::*lacZ*	Cip	Ceft	Azt
LAR005	Insertion of TCCA after A_314_		479 ± 24	2.0	8.0	32
LAR005Δ*cpxR*			232 ± 11	1.0	4.0	16
LAR023	G_352_AG→TAG		537 ± 16	4.0	8.0	32
LAR023Δ*cpxR*			264 ± 15	2.0	4.0	16
LAR048	Deletion of C_114_		484 ± 28	1.0	8.0	32
LAR048Δ*cpxR*			219 ± 8	0.5	4.0	16

## Discussion

In this work, we applied comparative genomic analysis to illuminate the regulatory networks responsible for modulating RND efflux pump expression in *P*. *aeruginosa*. Similar comparative genomic analysis has been performed to identify novel regulons based on conserved DNA motifs on the promoter regions of potential target genes as binding sites of global regulators [7, 48, 49]. With the accumulation of whole-genome sequencing and transcriptomic data, comparative genomic analysis has become a powerful approach for identifying common or species-specific genetic regulatory networks among different species.

Our work has demonstrated a novel regulatory linkage between CpxR and MexAB-OprM, an important efflux pump in *P*. *aeruginosa*. The significance of this regulatory linkage is several-fold: first, the regulatory influence of CpxR on RND efflux pump expression, even for pumps within the same orthologous group, could be very different among bacterial species. The direct regulatory influence of CpxR on expression of the *mexAB-oprM* orthologous operon is unique in *P*. *aeruginosa* (see Fig 1 and Table 1). Furthermore, it has been observed that the *mdtABCD* operon (encoding a RND efflux pump) possesses CpxR binding sites on its promoter region, while its expression is negatively regulated by CpxR in *E*. *coli* under conditions that activate the Cpx system [42]. In contrast, in this work, we demonstrate that the *muxABC-opmB* operon, an orthologue of the *mdtABCD* operon from *E*. *coli* (see S2 Table), is directly activated by CpxR in *P*. *aeruginosa* (see Table 1). Second, given that CpxR is involved in positive regulation of RND efflux pump expression in *Vibrio cholerae* [36] and *P*. *aeruginosa*, bioinformatics studies predict that VexAB/VexGH from *V*. *cholerae* [36] and MuxABC/MexAB from *P*. *aeruginosa* belong to different orthologous groups (for details, see S2 Table). Taken together, our observations suggest that the involvement of CpxR in regulating RND efflux pump expression may be evolutionarily divergent among bacterial species.

Incorporation of the MexAB-OprM efflux pump into the CpxR regulon reinforces the physiological importance of this efflux pump in *P*. *aeruginosa*. Indeed, the MexAB-OprM efflux pump plays profound physiological roles in addition to its role in antibiotic resistance in *P*. *aeruginosa*, including quorum sensing signal trafficking [50] and mediating bacterial virulence in hosts [51–53]. These functions of MexAB-OprM suggest that the regulatory linkage between CpxR and MexAB-OprM might have other purposes in addition to its role in antibiotic resistance in *P*. *aeruginosa*.

In this work, we have demonstrated that CpxR plays an important role in multidrug resistance by directly activating expression of *mexAB-oprM* in *nalB*-type *P*. *aeruginosa* isolates generated in the laboratory and collected in the clinic. Direct regulation of *mexAB-oprM* by CpxR suggests the existence of multiple pathways through which the expression level of the MexAB-OprM efflux pump might be elevated in *P*. *aeruginosa*. The Cpx system is involved in the cellular response to misfolded membrane proteins in *E*. *coli* [22] and *V*. *cholerae* [54]. Recently, several works have demonstrated the existence of a resistome in the genome of *P*. *aeruginosa* consisting of a broad array of genes belonging to different functional families, which give rise to decreased susceptibility to antibiotics when they are mutated [55–59]. Interestingly, a number of genes encoding membrane proteins belong to the resistome [58,59]. Future studies should assess whether CpxR is activated in response to misfolded membrane proteins as a means of determining whether it contributes to resistome expression in *P*. *aeruginosa*. Unlike the *cpxRA* operons in *E*. *coli* and *V*. *cholerae*, the *cpxR* locus is not directly linked to the sensor kinase gene locus in the genome of *P*. *aeruginosa*. Characterization of the signalling mechanism underlying the newly identified regulatory linkage between CpxR and MexAB-OprM, as well as identification of candidate CpxA sensors in *P*. *aeruginosa*, is underway. The combined effects of various signals mediated by multiple regulators, including CpxR and MexR, on MexAB-OprM expression will be understood in a broader physiological context in the near future.

## Materials and Methods

### Comparative genomic analysis

For the determination of putative orthologous proteins, a primary BLASTP search in a given genome was conducted for the gene with the highest similarity. Next, additional searches for conserved functional motifs were conducted based on the literature when appropriate.

Sequence retrieval and BLASTP searches related to whole-genome sequenced *Pseudomonas* species/strains were conducted using the Pseudomonas database (http://www.pseudomonas.com)[39], whereas other species/strains were analysed using the KEGG database (http://www.genome.jp/kegg/). Sequence similarity was determined using the online Pairwise alignment tool (http://www.ebi.ac.uk/Tools/psa/emboss_water/).

The intergenic regions containing the promoters of orthologous RND efflux pump operons were retrieved from 15 whole-genome-sequenced *Pseudomonas* species: *P*. *aeruginosa* PAO1, *P*. *aeruginosa* PA14, *P*. *fluorescens* Pf0-1, *P*. *fluorescens* SBW25, *P*. *syringae* pv. *phaseolicola* 1448A, *P*. *syringae* pv. *syringae* B728a, *P*. *entomophila*, *P*. *putida* GB-1, *P*. *putida* KT2440, *P*. *mendocina* ymp, *P*. *mendocina* NK-01, *P*. *stutzeri* A1501, *P*. *stutzeri* ATCC 17588, *P*. *brassicacearum*, and *P*. *fulva*.

For the inter-species analysis, conserved DNA motifs were obtained by alignment of the intergenic regions preceding the orthologous RND efflux pump operons using the MEME suite of online software [60]. For the intra-species analysis for putative CpxR binding sites, an online DNA motif search programme (http://www.pseudomonas.com/replicon/setmotif) was used to scan the entire genome of *P*. *aeruginosa* PA14 entering GTAAAN(4,8)GTAAA as the query form.

### Deletion of gene loci in *P*. *aeruginosa* strains

Generation of gene-locus-deleted *P*. *aeruginosa* strains was conducted using a method described previously [61]. For each gene, an upstream region including the start codon (longer than 500 bp) and a downstream region containing the stop codon (longer than 500 bp) were PCR-amplified and linked together. The resulting fragment was cloned into the suicide plasmid pEX18Tc. A fragment containing the *FRT* gentamicin-resistance (Gm) cassette from plasmid pPS856 was then inserted between flanking regions of the plasmid. The gene locus of each *P*. *aeruginosa* strain was then replaced with the plasmid by double-crossover homologous recombination. The Gm-resistance marker in the chromosome was removed by introducing plasmid pFLP2, which carries the Flp recombinase gene. Correct deletion in the constructed mutant was verified by PCR using primers that bound to flanking chromosomal regions of the fragments cloned into pEX18Tc. All DNA primers used in this study are listed in S3 Table.

### Construction of promoter-*lacZ* reporter gene fusion products and β-galactosidase assays

The promoter region of each gene was PCR-amplified and TA-cloned into the pEASY-T1 vector (TransGen, China). Site-directed mutagenesis was performed using a protocol described previously [62]. Disruption of the CpxR binding site on the *mexA* promoter (*mexA*p_M1_) was performed by altering the 5′-GTAAACCTAATGTAAA-3′ sequence to 5′-GTAAACCTAATACAAA-3′. Exclusion of the entire proximal promoter of *mexA* (up to 162 bp from the *mexA* ATG codon) was performed by PCR-amplifying the distal promoter only (*mexA*p_M2_). Disruption of the -10 region of the *mexA* distal promoter (*mexA*p_M3_) was performed by altering the 5′-TATTTT-3′ sequence to 5′-TGTTTT-3′. Once confirmed by sequencing, the promoter regions were subcloned into the broad-host, low-copy-number plasmid pMP190 [63]. The resulting plasmids were introduced into *Pseudomonas* strains by conjugal transfer from *E*. *coli* donor strain ST18 [64]. For the β-galactosidase assays, cells were grown overnight in Muller Hinton broth (Oxoid) supplemented with appropriate antibiotics, after which they were diluted 1:50 in 5 mL of fresh medium in 50-mL culture flasks at 37°C (30°C for *P*. *putida* KT2440) with mixing at 150 rpm. Cells were recovered during the logarithmic growth phase (OD_600_ = 0.5–1.2). β-galactosidase assays were performed as described by Miller [65]. The results are expressed as the mean values from two independent experiments with triplicate samples.

### Construction of *in trans* CpxR expression plasmids and purification of His_6_-CpxR

In order to control *in trans* CpxR expression, the *lacI* ^q^-*tac*P region was PCR-amplified using the pME6032 plasmid [66] as a template and cloned into broad-host plasmid pBBR1MCS5 to replace the original constitutively expressed *lac* promoter [67]. Next, the CpxR and CpxR^D52A^ *in trans* expression systems (pCpxR and pCpxR^D52A^) were constructed using the altered plasmid. IPTG (0.2 mM) was added to induce CpxR overexpression. It was noted that the basal level of CpxR expression in the absence of IPTG was sufficient to complement the *cpxR* deletion mutants in *P*. *aeruginosa*.

The plasmid used to express the N-terminal His_6_-tagged CpxR proteins was constructed by PCR-amplifying the CpxR coding sequence and cloning it into pET28a (Novagen). The plasmid was transformed into *E*. *coli* expression host strain BL21(DE3) and grown to an OD_600_ of 0.8 at 37°C with vigorous shaking in 1 L of LB medium containing kanamycin (50 μg/mL). The cells were then induced with 1 mM IPTG and allowed to express overnight at 22°C, after which they were harvested by centrifugation. The resulting pellet was resuspended in 10 mL of pre-cooled buffer A (20 mM Tris-HCl, 200 mM NaCl, 1 mM imidazole, pH 8.0) and centrifuged at 4°C for 10 minutes at 3,500 rpm, after which the pellet was resuspended in 60 mL of pre-cooled buffer A. The cells were disrupted by sonication at 180 W for 8 minutes. The debris and membranes were removed by centrifugation at 4°C for 60 minutes at 15,000 rpm. The soluble fraction was passed through a 0.2-μm filter and loaded onto a 5-mL nickel column which was previously washed with 10 column volumes of ddH_2_O and equilibrated with 10 column volumes of buffer A. CpxR proteins were eluted with a mixture of buffer A and buffer B (20 mM Tris-HCl, 200 mM NaCl, 500 mM imidazole, pH 8.0), in which the proportion of buffer B was gradually increased from 0% to 100%. The flow speed was 1 mL/min during the elution process. The protein was collected when its protein peak appeared. The CpxR protein solution was desalted and concentrated to a final volume of 1.5 mL in buffer C (20 mM Tris-HCl, 200 mM NaCl, pH 8.0). The concentration of CpxR protein was determined by the Bradford method. CpxR protein was stored in buffer C supplemented with 50% glycerol at -80°C.

### EMSAs

Purified N-terminal His-tagged CpxR proteins were phosphorylated using acetyl phosphate (AP) as previously described [68]. Briefly, 1.6 μM of purified CpxR was incubated with 50 mM AP in a reaction buffer containing 100 mM Tris-HCl (pH 7.4), 10 mM MgCl_2_, and 125 mM KCl at 30°C for 2 hours. The mobility shift assay was carried out using the 2^nd^ generation DIG Gel Shift Kit (Roche). Briefly, 165 bp DNA fragments of the promoter region of *mexA*p and *mexA*p_M1_, 140 bp DNA fragments of the promoter region of *cpxP*p were PCR-amplified, after which 150 nM of purified PCR product was DIG-labelled according to the manufacturer’s instructions. The binding reaction was carried out with different concentrations of phosphorylated CpxR (as great as 160 nM) and 0.2 nM DIG-labelled DNA fragments at 37°C for 30 min. The samples were separated by electrophoresis on 6% native polyacrylamide gels, transferred to Hybond-N blotting membranes (Amersham), and visualized by chemiluminescence.

### DNase I footprinting assay

The promoter region of *mexA* (165 bp) was TA-cloned into the pEASY-Blunt-simple vector (TransGen, China). For the preparation of fluorescent FAM labelled probes, the promoter region of *mexA* was PCR-amplified with Dpx DNA polymerase (TOLO Biotech, Shanghai) from the above-mentioned plasmid using primer pairs M13F-47(FAM)/M13R-48 and M13R-48(FAM)/M13F-47. The FAM-labelled probes (322 bp) were purified by the Wizard SV Gel and PCR Clean-Up System (Promega, USA) and quantified using the Take3 Micro-Volume Plate function of BioTek Synergy Neo Multi-Mode Reader.

DNase I footprinting assays were performed as previously reported [69]. Briefly, 400 ng of the probe (final concentration of 50 nM) was incubated with 1.5 μg of phosphorylated CpxR (final concentration of 1.5μM) in a total volume of 40 μL. After incubation for 30 min at 25°C, 10 μL of a solution containing approximately 0.015 units of DNase I (Promega) and 100 nmol of freshly prepared CaCl_2_ was added. Following incubation for 1 min at 25°C, the reaction was stopped by adding 140 μL of DNase I stop solution (200 mM unbuffered sodium acetate, 30 mM EDTA, and 0.15% SDS). Samples were extracted with phenol/chloroform and precipitated with ethanol, after which the pellets were dissolved in 30 μL of Milli-Q water. The preparation of the DNA ladder, electrophoresis, and data analysis were performed as described before [69], except that a GeneScan-LIZ500 size standard (Applied Biosystems) was used.

### Quantitative real-time PCR assay

An overnight culture (approximately 16 h) was diluted 1:100 in Mueller–Hinton broth and grown to the logarithmic growth phase (OD_600_ = 0.4~0.6). Total RNA was extracted from 500 μL of cultured cells using the RNAprep Bacterial Kit (TianGen, China). Residual genomic DNA was digested by RQ1 RNase-Free DNase (Promega, USA). RNA samples were quantified using the Take3 Micro-Volume Plate function of a BioTek Synergy Neo Multi-Mode Reader. cDNA was synthesized from 1 μg of total RNA using TransScript cDNA Synthesis SuperMix (TransGen, China) according to the following procedure: after annealing of the RNA sample and the random hexamer primer for 5 min at 65°C, reverse transcription was carried out for 2 min at 25°C and 55 min at 42°C, followed by reverse transcriptase inactivation for 5 min at 70°C. An Opticon2 Real-time PCR system (Bio-Rad, Hercules, CA, USA) and SuperReal Premix SYBR Green Plus (TianGen, China) were used to perform quantitative PCR on a 1-μL sample of diluted cDNA (1:10) according to the following procedure: one denaturation cycle for 15 min at 94°C and 40 amplification cycles for 10 s at 94°C, annealing for 20 s at 60°C, extension for 20 s at 72°C. Control samples without reverse transcriptase confirmed the absence of contaminating DNA in any of the samples. The housekeeping gene *rpsL* was used as the internal reference gene. Relative expression of *mexB* was calculated according to the 2^−ΔΔ*CT*^ method [70] from three independent experiments. Primers for *mexB* and *rpsL* were designed as previously reported [17, 71]

### Western blot assay

For the western blot detection of CpxR protein in PA14Δ*cpxR* containing pCpxR or pCpxR^D52A^, an overnight culture was diluted 1:100 in Mueller–Hinton broth and grown to the logarithmic growth phase (OD_600_ = 0.8–1.2). Total protein was extracted using a Bacterial Protein Extraction Kit (CWBiotech, China). Next, 5 μg of total protein and 10 ng of purified His-tagged CpxR were resolved in 10% SDS-polyacrylamide gels and transferred electrophoretically to PVDF membranes (Millipore, USA). Electrophoretic transfer of proteins was carried out for 1 h at 4°C with 200 mA of constant current. The blotted membranes were subsequently blocked in phosphate-buffered saline containing 0.1% (vol/vol) Tween-20 (PBST) and 5% (wt/vol) skim milk (Difco) for 60 min. The membranes were incubated with primary anti-CpxR rabbit polyclonal antibodies (1:10000) in PBST containing 5% (wt/vol) skim milk at 37°C for 60 min, after which they were washed three times (5 min each) with PBST and three times (5 min each) with PBS. The membranes were incubated with secondary goat anti-rabbit IgG antibodies conjugated to horseradish peroxidase (HRP) in PBST containing 5% (wt/vol) skim milk, after which they were washed three times (5 min each) with PBST and three times (5 min each) with PBS. All washes were carried out at room temperature with agitation. Substrates for HRP were obtained from the Amersham ECL Western Blotting Detection Kit (Amersham) and used according to the manufacturer’s instructions. The enzymatic activity of HRP was detected using a 4200 Chemiluminescence Analyzer (Tanon, China).

To allow detection of MexA protein by western blotting, cell membrane proteins were isolated by ultracentrifugation. Briefly, an overnight culture was diluted 1:100 in Mueller–Hinton broth and grown to the logarithmic growth phase (OD_600_ = 0.4–0.6). After sonication, total cell membrane protein was extracted from 40 mL of cultured cells using a Bacterial Membrane Protein Isolation Kit (Tiandz, Inc., China) according to the manufacturer’s instructions. The ultracentrifuged cell membrane protein pellets were resuspended in 100 μL of H_2_O. The concentrations of the membrane proteins were quantified using the Take3 Micro-Volume Plate function of a BioTek Synergy Neo Multi-Mode Reader. SDS-PAGE and immunoblotting for cell membrane proteins (10 μg of each sample) were performed as described above, except for the following: SDS (0.1% (wt/vol)) was included in the blotting buffer, the transfer was carried out for 16 h at 4°C with 25 mA constant current, and anti-MexB rabbit polyclonal antiserum (1:10000) was used as the primary antibody. Band intensity was quantified in three independent experiments using Image-pro Plus version 6.0.

### Antibiotic susceptibility test

The MIC of each antibiotic was determined on Muller Hinton agar by the two-fold dilution method. Mueller-Hinton agar plates containing serial twofold dilutions of each antibiotic (from 0.03125 to 32 μg/mL for ciprofloxacin and ofloxacin, from 0.125 to 128 μg/mL for the other antibiotics) were prepared. Overnight bacterial cultures were diluted 1:100 in fresh Mueller-Hinton broth, grown to the mid-logarithmic phase (OD600 of 0.4–0.6), harvested, and washed in PBS. The Mueller-Hinton agar plates were spotted with 3 μL of the diluted bacterial suspensions (approximately 10^4^ cfu). The MIC was defined as the concentration at which bacterial growth was completely inhibited after incubation at 37°C for 24 hours. Ciprofloxacin, ofloxacin, and amikacin were purchased from Bio Basic Inc. Ceftazidime was purchased from Sigma-Aldrich. Cefsulodin was purchased from TOKU-E (Japan). Aztreonam was purchased from Selleck.

### Generation of ofloxacin-cefsulodin resistant isolates in the laboratory

*P*. *aeruginosa* PA14 cells (approximately 4 × 10^9^ cells) grown overnight in LB broth medium were plated on LB agar containing 1.2 μg/mL ofloxacin and 2.4 μg/mL cefsulodin. After incubation at 37°C for 72 hours, resistant colonies appeared at a frequency of approximately 10^−7^.

### Multidrug resistant *P*. *aeruginosa* clinical isolates

Fifty independent *P*. *aeruginosa* clinical isolates (LAR001–LAR050) characterized as amikacin-sensitive (MIC≤ 2.0 μg/mL), ciprofloxacin-resistant (MIC range, 1.0–16 μg/mL), and ceftazidime-resistant (MIC range, 4.0–64 μg/mL) were obtained from the Department of Microbiology, Chinese People’s Liberation Army General Hospital (Beijing, China).

## Supporting Information

S1 Fig Identification of conserved DNA motifs. Conserved DNA motifs identified on the promoters of *mexA* (A) and *cpxP* (B) orthologues in *Pseudomonas* species. The upstream regions of orthologous genes from 15 whole-genome-sequenced *Pseudomonas* species were aligned using the MEME suite of online software. High probability (≥ 70%) nucleotides are highlighted in grey in the alignment. Consensus DNA motifs were deduced.
(TIF)

S2 Fig Comparison of the amino acid sequences of CpxP proteins. The amino acid sequences of the CpxP proteins from *P*. *aeruginosa* PA14 (CpxP_Pa) and *E*. *coli* (CpxP_Ec) were aligned using ClustalX software. The two conserved LTXXQ motifs are boxed in red.
(TIF)

S3 Fig Western blot analysis of CpxR and CpxR^D52A^ expression in PA14Δ*cpxR* strains containing the plasmids harbouring the respective genes. Protein samples were resolved in 10% SDS-polyacrylamide gels, transferred to PVDF membranes, and immunoblotted with anti-CpxR polyclonal antibodies. Lane 1, 10 ng of purified His-tagged CpxR; lanes 2, 5 μg of total protein from PA14Δ*cpxR* containing pCpxR; lane 3, 5 μg of total protein from PA14Δ*cpxR* containing pCpxR^D52A^; lane 4, 5 μg of total protein from PA14Δ*cpxR* containing empty vector.
(TIF)

S4 Fig **Direct binding of CpxR to the target promoter regions *in vitro* illustrated by EMSAs** with purified His-tagged CpxR and DIG-labelled DNA fragments of *cpxP*p (A), *mexA*p (B) or CpxR binding-site-mutated *mexA*p_M1_ (C). Phosphorylated CpxR protein (0, 20, 40, 80, and 160 nM) and DIG-labelled DNA fragments (0.2 nM) were added to the binding reaction (lanes 1–5). For the competition control (lane 6), an excess amount of unlabelled competitor DNA (20 nM) was added to the reaction mixture, which had the same composition as that of lane 5. For the unphosphorylated control (lane 7), 160 nM of unphosphorylated CpxR protein was added.
(TIF)

S1 Table Intergenic regions containing consensus CpxR binding sites in the genome of *P*. *aeruginosa* PA14. (DOCX)

S2 Table Orthologue prediction among 12 RND pumps of *P*. *aeruginosa* according to sequence similarity. (DOCX)

S3 Table Sequences of DNA primers used in this study. (DOCX)
