# Supplementary figures and images for: CpxR Activates MexAB-OprM Efflux Pump Expression and Enhances Antibiotic Resistance in Both Laboratory and Clinical nalB-Type Isolates of Pseudomonas aeruginosa
Source: PLoS Pathog. 2016 Oct 13;12(10):e1005932. doi: 10.1371/journal.ppat.1005932 (PMC5063474; doi:10.1371/journal.ppat.1005932)

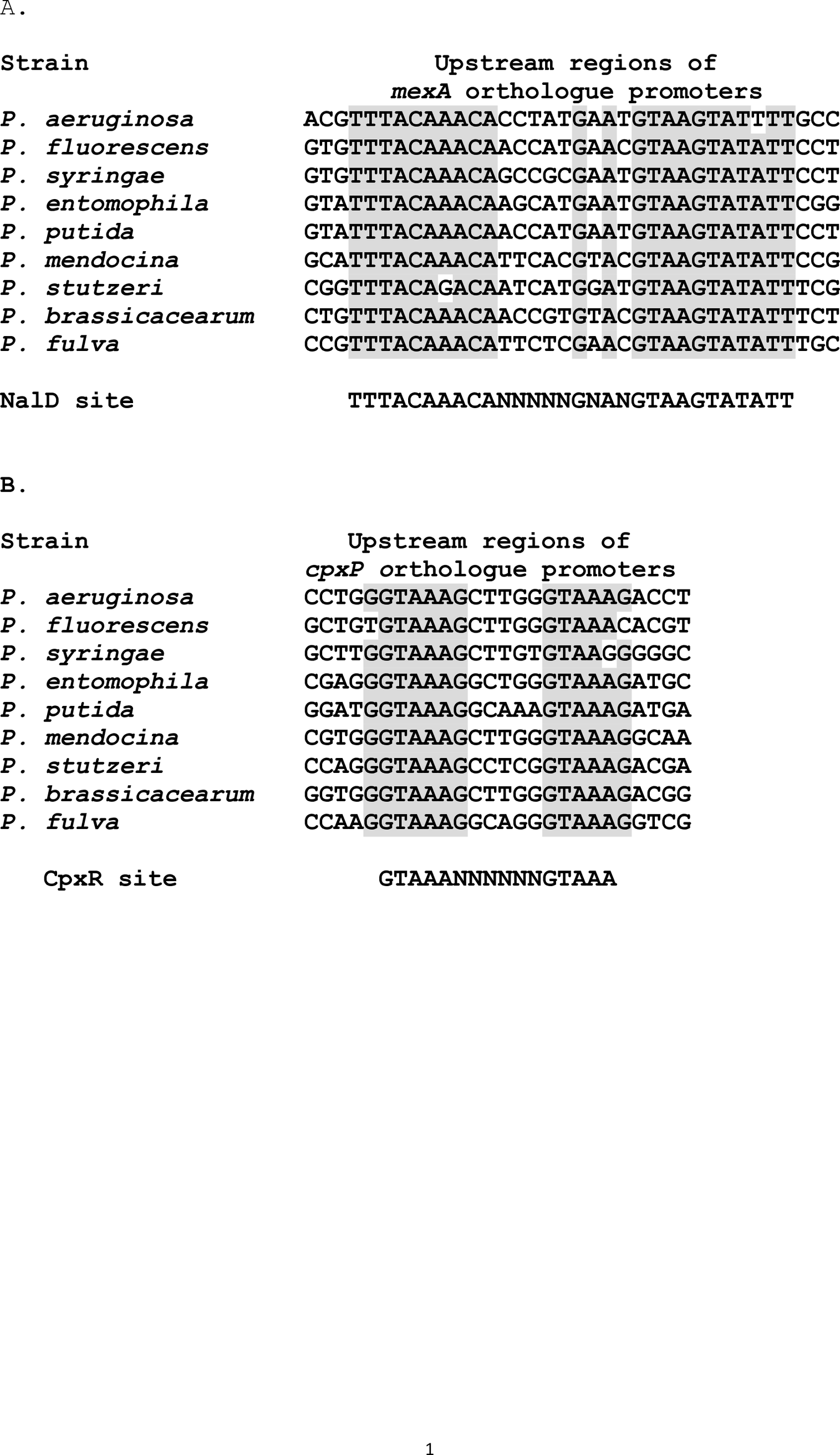

Supplement: S1 Fig — Conserved DNA motifs identified on the promoters of mexA (A) and cpxP (B) orthologues in Pseudomonas species. The upstream regions of orthologous genes from 15 whole-genome-sequenced Pseudomonas species were aligned using the MEME suite of online software. High probability (≥ 70%) nucleotides are highlighted in grey in the alignment. Consensus DNA motifs were deduced. (TIF) [file ppat.1005932.s001.tif]

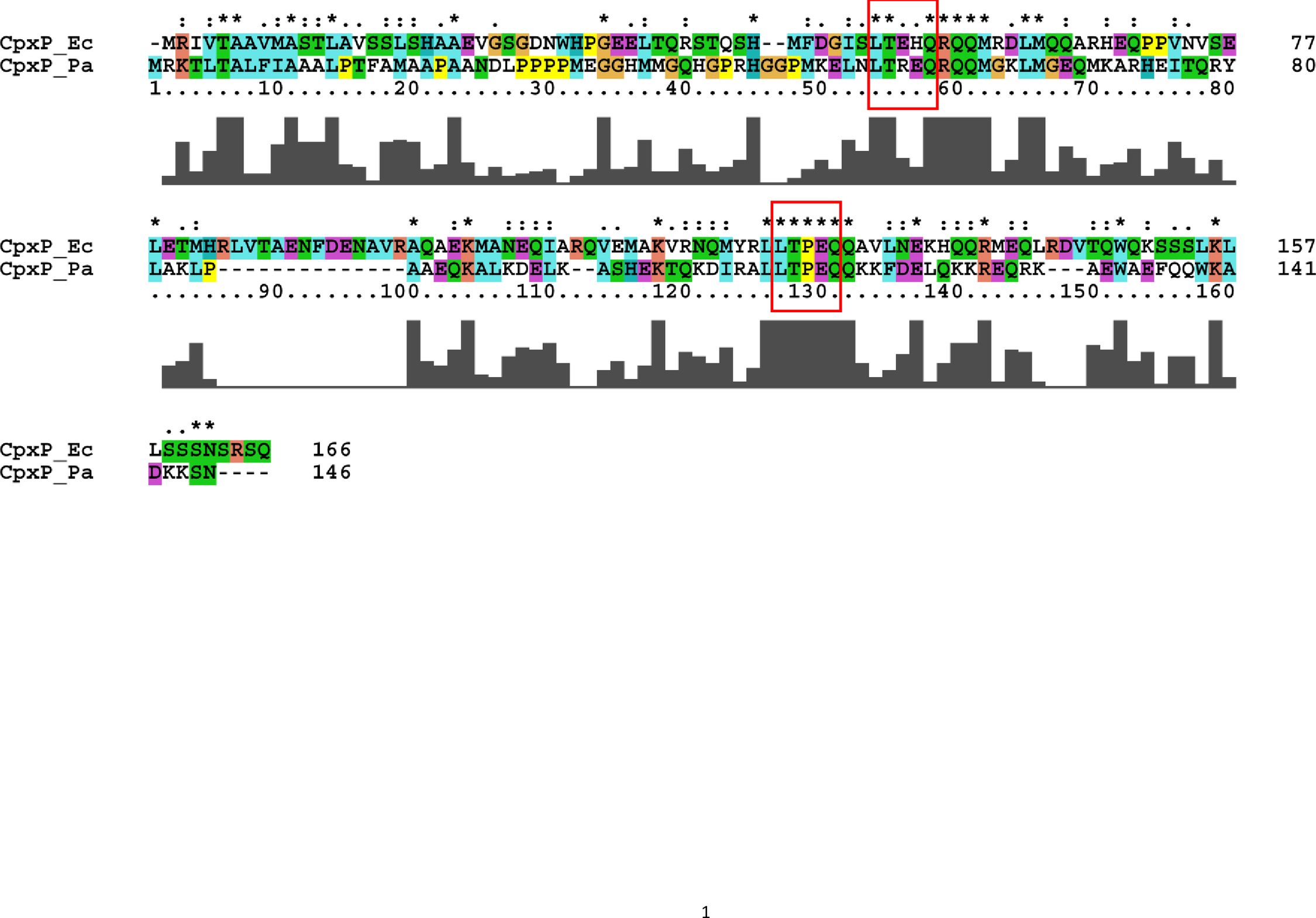

Supplement: S2 Fig — The amino acid sequences of the CpxP proteins from P. aeruginosa PA14 (CpxP_Pa) and E. coli (CpxP_Ec) were aligned using ClustalX software. The two conserved LTXXQ motifs are boxed in red. (TIF) [file ppat.1005932.s002.tif]

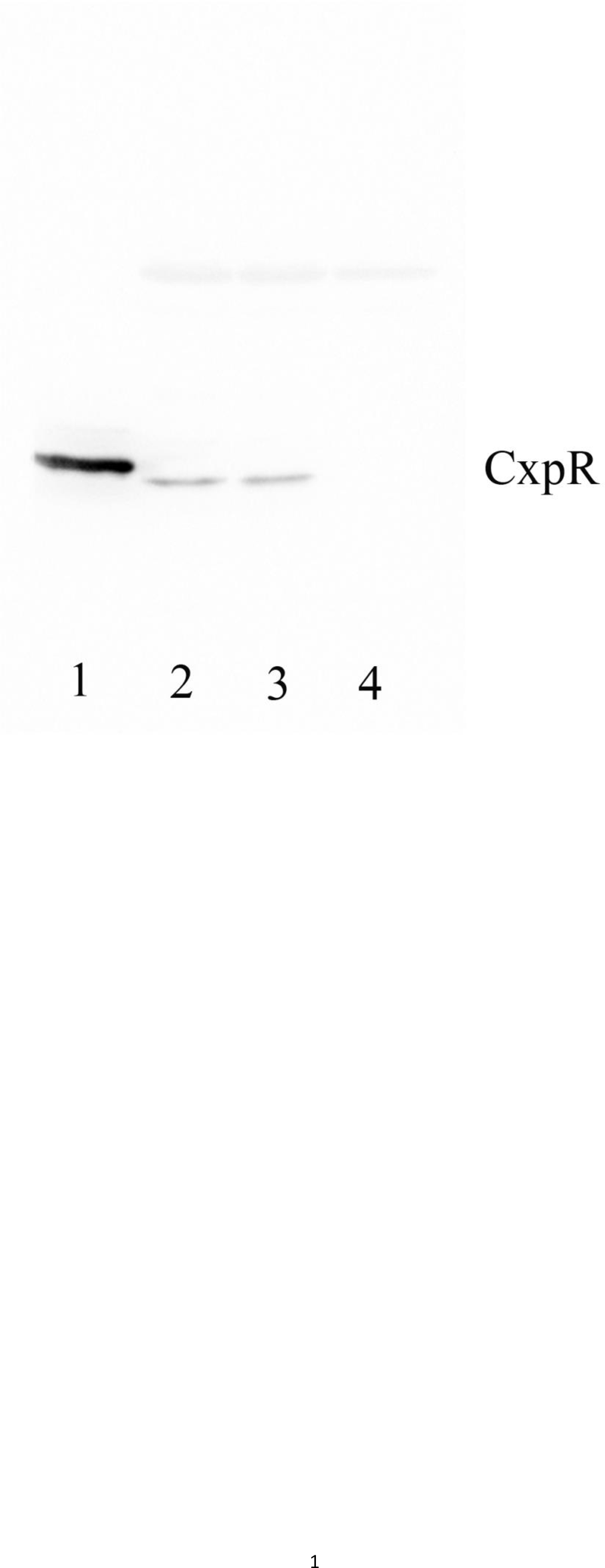

Supplement: S3 Fig — Protein samples were resolved in 10% SDS-polyacrylamide gels, transferred to PVDF membranes, and immunoblotted with anti-CpxR polyclonal antibodies. Lane 1, 10 ng of purified His-tagged CpxR; lanes 2, 5 μg of total protein from PA14ΔcpxR containing pCpxR; lane 3, 5 μg of total protein from PA14ΔcpxR containing pCpxRD52A; lane 4, 5 μg of total protein from PA14ΔcpxR containing empty vector. (TIF) [file ppat.1005932.s003.tif]

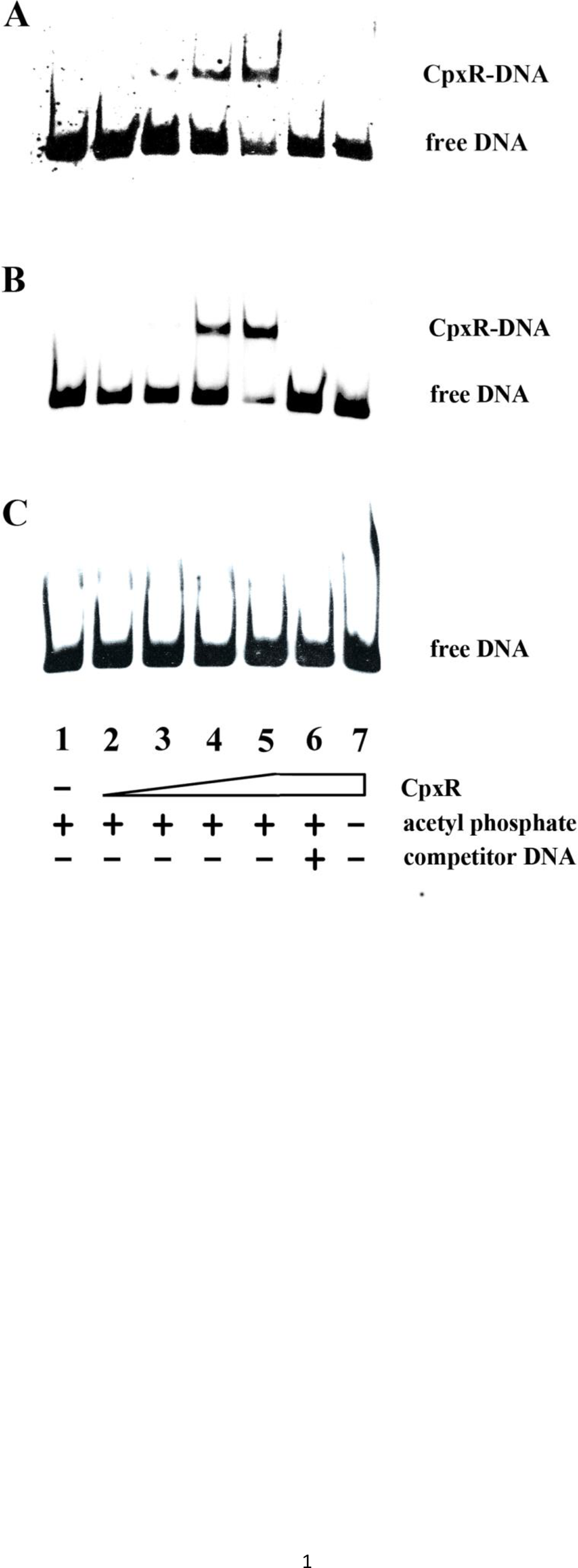

Supplement: S4 Fig — Direct binding of CpxR to the target promoter regions in vitro illustrated by EMSAs with purified His-tagged CpxR and DIG-labelled DNA fragments of cpxPp (A), mexAp (B) or CpxR binding-site-mutated mexApM1 (C). Phosphorylated CpxR protein (0, 20, 40, 80, and 160 nM) and DIG-labelled DNA fragments (0.2 nM) were added to the binding reaction (lanes 1–5). For the competition control (lane 6), an excess amount of unlabelled competitor DNA (20 nM) was added to the reaction mixture, which had the same composition as that of lane 5. For the unphosphorylated control (lane 7), 160 nM of unphosphorylated CpxR protein was added. (TIF) [file ppat.1005932.s004.tif]
