# Supplementary material for: CpxR Activates MexAB-OprM Efflux Pump Expression and Enhances Antibiotic Resistance in Both Laboratory and Clinical nalB-Type Isolates of Pseudomonas aeruginosa
Source: PLoS Pathog. 2016 Oct 13;12(10):e1005932. doi: 10.1371/journal.ppat.1005932 (PMC5063474; doi:10.1371/journal.ppat.1005932)
Supplement: S1 Table — (DOCX) [file ppat.1005932.s005.docx]

S1 Table. Intergenic regions containing consensus CpxR binding sites in the genome of *P. aeruginosa* PA14

| Orientation | Matching sequence | BP coordinates of match | Left gene | Distance to left gene | Right gene | Distance to right gene |
| --- | --- | --- | --- | --- | --- | --- |
| + | GTAAACCTAATGTAAA | 486513-486528 | *mexR* | 13 | *mexA* | 247 |
| - | GTAAAGAACGTAAA | 1889005-1889018 | *PA14_21720* | 30 | *PA14_21730* | 54 |
| + | GTAAAAATGGAGTAAA | 858955-858970 | *fpvB* | 112 | *dkgB* | 216 |
| - | GTAAAGAATGTAAA | 5219675-5219688 | *PA14_58570* | 92 | *PA14_58580* | 113 |
| + | GTAAAGCTTCAGTAAA | 1272927-1272942 | *PA14_14990* | 145 | *PA14_15000* | 21 |
| - | GTAAACGCCGGGTAAA | 1080354-1080369 | *PA14_12570* | 327 | *PA14_12590* | 40 |
| + | GTAAAGCTTGGGTAAA | 1978642-1978657 | *PA14_22740 (cpxP)* | 82 | *cpxR* | 32 |
| + | GTAAAGCCTGTGTAAA | 4852464-4852479 | *PA14_54720* | 38 | *PA14_54730* | 1 |
| - | GTAAATAGAGCGTAAA | 5217238-5217253 | *PA14_58560* | 132 | *PA14_58570* | 69 |
| + | GTAAAGCAAGGGTAAA | 2771950-2771965 | *PA14_31850* | 152 | *muxA* | 90 |
| - | GTAAAGGCGGAGTAAA | 1550686-1550701 | *PA14_18060* | 64 | *PA14_18070* | 104 |
