# Supplementary material for: CpxR Activates MexAB-OprM Efflux Pump Expression and Enhances Antibiotic Resistance in Both Laboratory and Clinical nalB-Type Isolates of Pseudomonas aeruginosa
Source: PLoS Pathog. 2016 Oct 13;12(10):e1005932. doi: 10.1371/journal.ppat.1005932 (PMC5063474; doi:10.1371/journal.ppat.1005932)
Supplement: S2 Table — (DOCX) [file ppat.1005932.s006.docx]

S2 Table. Orthologue prediction among 12 RND pumps of *P. aeruginosa* according to sequence similarity.

| **Strain MFP-RND^1^** | **MexA-MexB** | **MuxA-MuxB** | **MexV-MexW** | **MexC-MexD** |
| --- | --- | --- | --- | --- |
| ***E. coli* AcrA-AcrB** | **58%(71%)-70%(83%)**^2^ | 31%(50%)-29%(49%) | 28%(44%)-34%(55%) | 45%(67%)-50%(69%) |
| ***E. coli* MdtA-MdtB** | 24%(47%)-30%(51%) | **43%(61%)-67%(82%)** | 24%(44%)-31%(52%) | 30%(50%)-28%(48%) |
| ***V.cholerae* VexA-VexB** | 25%(43%)-31%(52%) | 23%(42%)-30%(51%) | **40%(60%)-49%(69%)** | 25%(43%)-31%(53%) |
| ***V.cholerae* VexG-VexH** | 22%(44%)-30%(50%) | 25%(48%)-30%(52%) | **32%(50%)-36%(58%)** | 25%(45%)-30%(52%) |

|  | **MexE-MexF** | **MexH-MexI** | **MexJ-MexK** | **MexM-MexN** |
| --- | --- | --- | --- | --- |
| ***E. coli* AcrA-AcrB** | 34%(51%)-41%(62%) | 26%(41%)-31%(51%) | 23%(39%)-25%(43%) | 32%(48%)-28%(49%) |
| ***E. coli* MdtA-MdtB** | 30%(49%)-31%(51%) | 27%(45%)-31%(52%) | 26%(45%)-23%(45%) | 36%(59%)-50%(68%) |
| ***V.cholerae* VexA-VexB** | 23%(40%)-33%(53%) | 36%(55%)-45%(68%) | 25%(41%)-23%(43%) | 26%(47%)-29%(50%) |
| ***V.cholerae* VexG-VexH** | 29%(48%)-31%(52%) | 28%(50%)-35%(55%) | 26%(47%)-26%(44%) | 24%(45%)-30%(52%) |

|  | **MexP-MexQ** | **MexX-MexY** | **CzcB-CzcA** | **TriB-TriC** |
| --- | --- | --- | --- | --- |
| ***E. coli* AcrA-AcrB** | 33%(53%)-40%(62%) | 41%(60%)-51%(71%) | 24%(44%)-24%(44%) | 24%(41%)-24%(42%) |
| ***E. coli* MdtA-MdtB** | 28%(45%)-31%(51%) | 28%(44%)-30%(52%) | 26%(43%)-27%(47%) | 25%(48%)-24%(45%) |
| ***V.cholerae* VexA-VexB** | 26%(43%)-32%(53%) | 20%(38%)-30%(53%) | 22%(45%)-25%(44%) | 23%(37%)-23%(44%) |
| ***V.cholerae* VexG-VexH** | 30%(48%)-31%(52%) | 23%(41%)-30%(52%) | 23%(42%)-26%(47%) | 23%(41%)-24%(44%) |

1 MFP, membrane fusion protein; RND, resistance-nodulation-division multidrug efflux transporter.

2 Sequence similarity was scored as the identities and positives (in parentheses) of the amino acid sequences of the MFP pairs and RND pairs using an online Pairwise alignment tool (<http://www.ebi.ac.uk/Tools/psa/emboss_water/>). The amino acid sequences were retrieved from the KEGG database (<http://www.genome.jp/kegg/>). The candidate orthologues with the highest similarity are marked in bold.
