# Supplementary material for: CpxR Activates MexAB-OprM Efflux Pump Expression and Enhances Antibiotic Resistance in Both Laboratory and Clinical nalB-Type Isolates of Pseudomonas aeruginosa
Source: PLoS Pathog. 2016 Oct 13;12(10):e1005932. doi: 10.1371/journal.ppat.1005932 (PMC5063474; doi:10.1371/journal.ppat.1005932)
Supplement: S3 Table — (DOCX) [file ppat.1005932.s007.docx]

S3 Table. Sequences of DNA primers used in this study

|  | 5′→3′ |
| --- | --- |
| **Primers for the promoter-*lacZ* fusion products** | |
| *cpxP* promoter F primer (*Xba*I) | GCTCTAGACATGCACGTCAGCAACCTGC |
| R primer (*Kpn*I) | GGGGTACCGTCTTGCGCATGGTGTTTCTCC |
| *muxA* promoter F primer (*Xba*I) | GCTCTAGACGTAGCGCAAGGAGTGGAATTC |
| R primer (*Kpn*I) | GGGGTACCGGTCGTTGGAGTCATGGTTC |
| *mexA* promoter F primer (*Xba*I) | GCTCTAGATGGTTTGGCCGAGTAAACCTAATG |
| R primer (*Kpn*I) | GGGGTACCAGCGTTGTCCTCATGAGCGAAAGC |
| *PP_4504* promoter F primer (*Xba*I) | GCTCTAGACCGCAGCCTGGACATGCATGTC |
| R primer (*Kpn*I) | GGGGTACCTTGCGCATGGTGTCTCTCCTTTC |
| *PP_3585* promoter F primer (*Xba*I) | GCTCTAGAAAGAACAGTGGGTTATC |
| R primer (*Kpn*I) | GGGGTACCAGCAGCAGGATCAGCAGG |
| *PP_1386 (ttgA)* promoter F primer (*Xba*I) | GCTCTAGAGTTGTTGAAGTGCCAGTAGATCG |
| R primer (*Kpn*I) | GGGGTACCTTGAATTGCATGAGGATCCTC |
| **Primers for *in trans* CpxR expression** | |
| *lacI*^q^-*tac*P F primer (*Nco*I) | TGCCATGGCTAACTTACATTAATTGCG |
| *lacI*^q^-*tac*P R primer (*Eco*RI) | CGAATTCTGTTTCCTGTGTG |
| *cpxR* F primer (*Eco*RI) | GGAATTCAGCGACGCGAATGGATCAATTG |
| *cpxR* R primer (*Bam*HI) | GGGGATCCTTGGGTAAAGACCTTGTGTCG |
| **Primers for N-terminal His-tagged CpxR expression** | |
| F primer (*Nde*I) | GGAATTCCATATGAGCGAACTGCTGTTGATCG |
| R primer (*Bam*HI) | GGGGATCCTTGGGTAAAGACCTTGTGTCG |
| **Primers for locus deletion** | |
| *cpxR* upstream region F primer (*Bam*HI) | GGGGATCCCGGCAAACGTTTTCGCACC |
| R primer (*Kpn*I) | CAGGTACCGAGCAGCTCGCAGAG |
| *cpxR* downstream region F primer (*Kpn*I) | GGGGTACCTGCGCAAGAAGCTCGGCAGC |
| R primer (*Eco*RI) | GGAATTCGGCCCACTCTGCCTTGC |
| *mexR* upstream region F primer (*Eco*RI) | GGAATTCGGTCGATCTGGTAGAGCTGCTGC |
| R primer (*Kpn*I) | GGGGTACCATTGGTTTGGCCGAGTAAAC |
| *mexR* downstream region F primer (*Kpn*I) | GGGGTACCCCAGCGACCAGCGCAGC |
| R primer (*Bam*HI) | GGGGATCCTTCGAATCCACCGAAGTGAAGG |
| *mexA* locus^†^ F primer (*Hind*III) | GATAAGCTTCAATACATGGACGTC |
| R primer (*Bam*HI) | CCGGATCCCCTGGCGCTGCACTTC |
| *muxA* locus^†^ F primer (*Eco*RI) | CGTAGCGCAAGGAGTGGAATTCGG |
| R primer (*Hind*III) | GCAAGCTTCGCCACCATCAGCAGGGTGGTC |
| **Primers for *in vitro* DNA binding probes** | |
| *mexA*p probe F primer | CATGCTGGAAGACCGCCATCAG |
| *mexA*p probe R primer | CATGGCCCATATTCAGAACCTG |
| *cpxP*p probe F primer | GGCTACTACTACAGCCACTGAGC |
| *cpxP*p probe R primer | CCTTTCTGGGTCCGGCCATTTAC |
|  |  |
|  |  |
| **Primers for real-time quantitative PCR** | |
| *mexB* F primer | GTGTTCGGCTCGCAGTACTC |
| *mexB* R primer | AACCGTCGGGATTGACCTTG |
| *rpsL* F primer | GCAAGCGCATGGTCGACAAGA |
| *rpsL* R primer | CGCTGTGCTCTTGCAGGTTGTGA |

^†^There are two *Sal*I sites in the coding regions of *mexA* and *muxA*; the sequences between these sites were replaced by the gentamicin resistance marker gene from pPS856.
